# Supplementary material for: Molecular Characterization and Pathogenicity of Alternaria spp. Associated with Black Rot of Sweet Cherries in Italy
Source: J Fungi (Basel). 2023 Oct 7;9(10):992. doi: 10.3390/jof9100992 (PMC10607616; doi:10.3390/jof9100992)
Supplement: Supplementary file 1 [file jof-09-00992-s001.zip › jof-2620457-supplementary.pdf]

Supplementary Materials

Table S1. Collection details and GenBank accession numbers of strains isolated from sweet cherries in Italy and reference strains included in this study for the phylogenetic analysis.

Species	Isolate	Location	Host	rpb2	Alt a 1	endoPG	OPA10-2
Alternaria alstroemeriae	CBS 118808; E.G.S. 50.116	USA	*Alstroemeria* sp.	KP124764	KP123845	KP123993	KP124601
A. alternata	CBS 107.27; ATCC 24463; QM 1736 (*A. citri*)	USA	*Citrus limonium*	KP124768	KP123849	KP123996	KP124604
A. alternata	CBS 103.33; E.G.S. 35.182; IHEM 3319 (*A. soliaegyptiaca*)	Egypt	soil	KP124770	KP123852	KP123999	KP124607
A. alternata	CBS 117.44; E.G.S. 06.190; VKM F-1870 (*A. godetiae*)	Denmark	*Godetia* sp.	KP124772	KP123854	KP124001	KP124609
A. alternata	CBS 102.47; E.G.S. 02.062 (*A. citri*)	USA	*Citrus sinensis*	KP124773	KP123855	KP124002	KP124610
A. alternata	CBS 174.52; E.G.S. 39.1613; IMI 068086; QM 1278	USA	*Anemone occidentalis*	DQ677964	KP123856	KP124003	KP124611
A. alternata	CBS 175.52; E.G.S. 35.1619; IMI 068085; QM 1277	USA	*Juncus mertensianus*	KC584445	KP123857	KP124004	KP124612
A. alternata	CBS 107.53; DSM 3187; IFO 5778 (*A. kikuchiana*)	Japan	*Pyrus pyrifolia*	KP124774	KP123858	KP124005	KP124613
A. alternata	CBS 612.72; DSM 62012 (*A. cinerariae*)	Germany	*Senecio cineraria*	KP124777	KP123861	KP124008	KP124615
A. alternata	CBS 620.83; ATCC 15052 (*A. tenuissima*)	USA	*Nicotiana tabacum*	KP124783	KP123868	KP124015	KP124622
A. alternata	CBS 195.86; E.G.S. 36.172; DAOM 185214 (A. angustiovoidea)	Canada	*Euphorbia esula*	KP124785	JQ646398	KP124017	KP124624
A. alternata	CBS 965.95; IMI 289679 (*A. tenuissima*)	India	*Triticum* sp.	KP124791	KP123872	KP124023	KP124629
A. alternata	CBS 918.96; E.G.S. 34.015; IMI 255532 (*A. tenuissima*)	UK	*Dianthus chinensis*	KC584435	AY563302	KP124026	KP124633
A. alternata	CBS 639.97; IMI 366417	Greece	*Helianthus annuus*	KP124795	KP123876	KP124028	KP124635
A. alternata	CBS 102595; E.G.S. 45.100 (*A. limoniasperae*)	USA	*Citrus jambhiri*	KC584408	AY563306	KP124029	KP124636
A. alternata	CBS 102599; E.G.S. 44.166 (*A. turkisafria*)	Turkey	*Minneola tangelo*	KP124798	KP123879	KP124032	KP124639
A. alternata	CBS 102600; E.G.S. 39.181; ATCC 38963	USA	*Citrus reticulata*	KP124799	KP123880	KP124033	KP124640
A. alternata	CBS 113054; CPC 4263 (*A. tenuissima*)	South Africa	*Malus domestica*	KP124814	KP123894	KP124047	KP124656
A. alternata	CBS 115069; CPC 4254 (*A. tenuissima*)	South Africa	*Malus domestica*	KP124815	KP123895	KP124048	KP124657
A. alternata	CBS 119115	Greece	*Prunus* sp.	KP124828	KP123909	KP124062	np*
A. alternata	CBS 120829	Greece	*Punica granatum*	KP124832	KP123912	KP124066	KP124675
A. alternata	CBS 121346; E.G.S. 45.056 (*A. turkisafria*)	South Africa	*Minneola tangelo*	KP124835	KP123914	KP124069	KP124678
A. alternata	CBS 121455; E.G.S. 50.078 (*A. broussonetiae*)	China	*Broussonetia papyrifera*	KP124838	KP123916	KP124072	KP124681
A. alternata	CBS 121492; HSAUP0207 (*Ulocladium cucumisis*)	China	*Cucumis melo*	KP124840	KP123918	KP124074	KP124683

A. alternata	CBS 126071 (*A. tenuissima*)	Namibia	soil	KP124846	KP123924	KP124080	KP124689
A. alternata	CBS 127671; E.G.S. 52.121 (*A. seleniiphila*)	USA	*Stanleya pinnata*	KP124851	KP123929	KP124085	KP124694
A. alternata	T6	Italy	*Prunus avium*	*OP899743*	*OP899663*	*OP899703*	OQ607083
A. alternata	GR3	Italy	*P. avium*	*OP899747*	*OP899667*	*OP899707*	OQ607084
A. alternata	GR13	Italy	*P. avium*	OP899750	OP899670	OP899710	OQ607085
A. alternata	Ch21	Italy	*P. avium*	*OP899767*	*OP899687*	*OP899727*	OQ607086
A. alternata	Ch23	Italy	*P. avium*	*OP899769*	*OP899689*	*OP899729*	OQ607087
A. alternata	Ch37	Italy	*P. avium*	*OP899773*	*OP899693*	*OP899733*	OQ607088
A. alternata	Ch43	Italy	*P. avium*	*OP899777*	*OP899697*	*OP899737*	OQ607089
A. arborescens SC	CBS 101.13; E.G.S. 07.022; QM1765 (*A. geophila*)	Switzerland	peat soil	KP124862	KP123940	KP124096	KP124705
A. arborescens SC	CBS 105.24; IHEM 3123 (*A. alternata*)	Unknown	*Solanum tuberosum*	KP124863	KP123941	KP124097	KP124706
A. arborescens SC	CBS 108.41; E.G.S. 44.087; ATCC 11892 (*A. alternata*)	Unknown	wood	KP124864	KP123942	KP124098	KP124707
A. arborescens SC	CBS 113.41; IHEM 3318 (*A. alternata*)	Unknown	*Schizanthus* sp.	KP124865	KP123943	KP124099	KP124708
A. arborescens SC	CBS 105.49 (*A. alternata*)	Italy	contaminant blood culture	KP124866	KP123944	KP124100	KP124709
A. arborescens SC	CBS 126.60; IMI 081622 (*A. maritima*)	UK	wood	KP124867	JQ646390	KP124101	KP124710
A. arborescens SC	CBS 750.68; LCP 68.1989 (*A. tenuissima*)	France	*Phaseolus vulgaris*	KP124868	KP123945	KP124102	KP124711
A. arborescens SC	CBS 102605; E.G.S. 39.128 (*A. arborescens*)	USA	*Solanum lycopersicum*	KC584377	AY563303	AY295028	KP124712
A. arborescens SC	CBS 109730 (*A. arborescens*)	USA	*Solanum lycopersicum*	KP124869	KP123946	KP124103	KP124713
A. arborescens SC	CBS 112633; CPC 4244 (*A. arborescens*)	South Africa	*Malus domestica*	KP124870	KP123947	KP124104	KP124714
A. arborescens SC	CBS 112749; CPC 4245 (*A. arborescens*)	South Africa	*Malus domestica*	KP124871	KP123948	KP124105	KP124715
A. arborescens SC	CBS 115189; CPC 4345 (*A. arborescens*)	South Africa	*Citrus clementina*	KP124872	KP123949	KP124106	KP124716
A. arborescens SC	CBS 115516; CPC 4247 (*A. arborescens*)	South Africa	*Malus domestica*	KP124873	KP123950	KP124107	KP124717
A. arborescens SC	CBS 115517; CPC 4246 (*A. arborescens*)	South Africa	*Malus domestica*	KP124874	KP123951	KP124108	KP124718
A. arborescens SC	CBS 116329 (*A. alternata*)	Germany	*Malus domestica*	KP124875	KP123952	KP124109	KP124719
A. arborescens SC	CBS 117587 (*A. alternata*)	Netherlands	*Brassica* sp.	KP124876	KP123953	KP124110	KP124720
A. arborescens SC	CBS 118389; E.G.S. 90.131 (*A. gaisen*)	Japan	*Pyrus pyrifolia*	KP124877	KP123954	KP124111	KP124721
A. arborescens SC	CBS 119544; E.G.S. 43.072 (*A. cerealis*)	New Zealand	*Avena sativa*	KP124878	KP123955	KP124112	KP124722
A. arborescens SC	CBS 119545; E.G.S. 48.130 (*A. senecionicola*)	New Zealand	*Senecio skirrhodon*	KP124879	KP123956	KP124113	KP124723

A. arborescens SC	CBS 123235 (*A. alternata*)	Denmark	human toenail	KP124880	KP123957	KP124114	KP124724
A. arborescens SC	CBS 123266 (*A. alternata*)	Denmark	human toenail	KP124881	KP123958	KP124115	KP124725
A. arborescens SC	CBS 123267 (*A. alternata*)	Denmark	human nail	KP124882	KP123959	KP124116	KP124726
A. arborescens SC	CBS 124281 (*A. arborescens*)	Denmark	*Triticum* sp.	KP124883	KP123961	KP124118	KP124728
A. arborescens SC	CBS 124282 (*A. arborescens*)	Denmark	*Hordeum vulgare*	KP124884	KP123962	KP124119	KP124729
A. arborescens SC	CBS 124283 (*A. tenuissima*)	Russia	*Oryza* sp.	KP124885	KP123963	KP124120	KP124730
A. arborescens SC	CBS 127263 (*A. alternata*)	Mexico	Human nasal infection	KP124886	KP123964	KP124121	KP124731
A. arborescens SC	D4	Italy	*P. avium*	OP899740	OP899660	OP899700	OQ607090
A. arborescens SC	T2	Italy	*P. avium*	OP899741	OP899661	OP899701	OQ607091
A. arborescens SC	T3	Italy	*P. avium*	OP899742	OP899662	OP899702	OQ607092
A. arborescens SC	T8	Italy	*P. avium*	OP899744	OP899664	OP899704	OQ607093
A. arborescens SC	T9	Italy	*P. avium*	OP899745	OP899665	OP899705	OQ607094
A. arborescens SC	GR1	Italy	*P. avium*	OP899746	OP899666	OP899706	OQ607095
A. arborescens SC	GR6	Italy	*P. avium*	OP899748	OP899668	OP899708	OQ607096
A. arborescens SC	GR8	Italy	*P. avium*	OP899749	OP899669	OP899709	OQ607097
A. arborescens SC	W2	Italy	*P. avium*	OP899751	OP899671	OP899711	OQ607098
A. arborescens SC	W6	Italy	*P. avium*	OP899752	OP899672	OP899712	OQ607099
A. arborescens SC	Q1	Italy	*P. avium*	OP899753	OP899673	OP899713	OQ607100
A. arborescens SC	X7	Italy	*P. avium*	OP899754	OP899674	OP899714	OQ607101
A. arborescens SC	Ch1	Italy	*P. avium*	OP899755	OP899675	OP899715	OQ607102
A. arborescens SC	Ch2	Italy	*P. avium*	OP899756	OP899676	OP899716	OQ607103
A. arborescens SC	Ch4	Italy	*P. avium*	OP899757	OP899677	OP899717	OQ607104
A. arborescens SC	Ch5	Italy	*P. avium*	OP899758	OP899678	OP899718	OQ607105
A. arborescens SC	Ch11	Italy	*P. avium*	OP899759	OP899679	OP899719	OQ607106
A. arborescens SC	Ch12	Italy	*P. avium*	OP899760	OP899680	OP899720	OQ607107
A. arborescens SC	Ch13	Italy	*P. avium*	OP899761	OP899681	OP899721	OQ607108
A. arborescens SC	Ch14	Italy	*P. avium*	OP899762	OP899682	OP899722	OQ607109
A. arborescens SC	Ch16	Italy	*P. avium*	OP899763	OP899683	OP899723	OQ607110
A. arborescens SC	Ch17	Italy	*P. avium*	OP899764	OP899684	OP899724	OQ607111
A. arborescens SC	Ch18	Italy	*P. avium*	OP899765	OP899685	OP899725	OQ607112

A. arborescens SC	Ch19	Italy	*P. avium*	OP899766	OP899686	OP899726	OQ607113
A. arborescens SC	Ch22	Italy	*P. avium*	OP899768	OP899688	OP899728	OQ607114
A. arborescens SC	Ch26	Italy	*P. avium*	OP899770	OP899690	OP899730	OQ607115
A. arborescens SC	Ch27	Italy	*P. avium*	OP899771	OP899691	OP899731	OQ607116
A. arborescens SC	Ch35	Italy	*P. avium*	OP899772	OP899692	OP899732	OQ607117
A. arborescens SC	Ch39	Italy	*P. avium*	OP899774	OP899694	OP899734	OQ607118
A. arborescens SC	Ch40	Italy	*P. avium*	OP899775	OP899695	OP899735	OQ607119
A. arborescens SC	Ch42	Italy	*P. avium*	OP899776	OP899696	OP899736	OQ607120
A. arborescens SC	Ch45	Italy	*P. avium*	OP899778	OP899698	OP899738	OQ607121
A. arborescens SC	Ch48	Italy	*P. avium*	OP899779	OP899699	OP899739	OQ607122
Alternaria betae-kenyensis	CBS 118810; E.G.S. 49.159; IMI 385709	Kenya	Beta vulgaris var. cicla	KP124888	KP123966	KP124123	KP124733
Alternaria burnsii	CBS 107.38; E.G.S. 06.185	India	*Cuminum cyminum*	KP124889	KP123967	KP124124	KP124734
Alternaria eichhorniae	CBS 489.92; ATCC 22255; ATCC 46777; IMI 121518	India	*Eichhornia crassipes*	KP124895	KP123973	KP124130	KP124740
Alternaria gaisen	CBS 632.93; E.G.S. 90.512	Japan	*Pyrus pyrifolia*	KC584399	KP123974	AY295033	KP124742
Alternaria gossypina	CBS 104.32	Zimbabwe	*Gossypium* sp.	KP124900	JQ646395	KP124135	KP124746
Alternaria iridiaustralis	CBS 118486; E.G.S. 43.014	Australia	*Iris* sp.	KP124905	KP123981	KP124140	KP124751
Alternaria jacinthicola	CBS 133751; MUCL 53159	Mali	*Eichhornia crassipes*	KP124908	KP123984	KP124143	KP124754
Alternaria longipes	CBS 121333; E.G.S. 30.051	USA	*Nicotiana tabacum*	KP124914	KP123990	KP124150	KP124761
Alternaria tomato	CBS 103.30	Unknown	*Solanum lycopersicum*	KP124915	KP123991	KP124151	KP124762
Alternaria nobilis	AC1	Japan	*Dianthus barbatus*	LC476798	LC481624	LC480952	np

*np: no product
